# Duration of Infectious Virus Shedding by SARS-CoV-2 Omicron Variant–Infected Vaccinees

**DOI:** 10.3201/eid2805.220197

**Published:** 2022-05

**Authors:** Kenichiro Takahashi, Masahiro Ishikane, Mugen Ujiie, Noriko Iwamoto, Nobumasa Okumura, Testsuro Sato, Maki Nagashima, Ataru Moriya, Michiyo Suzuki, Masayuki Hojo, Takayuki Kanno, Shinji Saito, Sho Miyamoto, Akira Ainai, Minoru Tobiume, Takeshi Arashiro, Tsuguto Fujimoto, Tomoya Saito, Masaya Yamato, Tadaki Suzuki, Norio Ohmagari

**Affiliations:** National Institute of Infectious Diseases, Tokyo, Japan (K. Takahashi, T. Kanno, S. Saito, S. Miyamoto, A. Ainai, M. Tobiume, T. Arashiro, T. Fujimoto, T. Saito, T. Suzuki);; National Center for Global Health and Medicine, Tokyo (M. Ishikane, M. Ujiie, N. Iwamoto, N. Okumura, T. Sato, M. Nagashima, A. Moriya, M. Suzuki, M. Hojo, N. Ohmagari);; Rinku General Medical Center, Osaka, Japan (M. Yamato)

**Keywords:** COVID-19, 2019 novel coronavirus disease, coronavirus disease, severe acute respiratory syndrome coronavirus 2, SARS-CoV-2, viruses, respiratory infections, zoonoses, Omicron, variant of concern, viral shedding, vaccines, Japan

## Abstract

To determine virus shedding duration, we examined clinical samples collected from the upper respiratory tracts of persons infected with severe acute respiratory syndrome coronavirus 2 Omicron variant in Japan during November 29–December 18, 2021. Vaccinees with mild or asymptomatic infection shed infectious virus 6–9 days after onset or diagnosis, even after symptom resolution.

The severe acute respiratory syndrome coronavirus 2 (SARS-CoV-2) variant of concern belonging to the Pango lineage B.1.1.529, known as the Omicron variant, has spread rapidly worldwide (*1*,*2*). Several reports describe high infectivity and transmissibility of Omicron (*3*,*4*). The clinical course and the duration of virus shedding based on cycle quantification (Cq) values among 11 Omicron-infected patients has been reported (*5*). However, the relationship between duration of virus shedding and infectivity of Omicron is unknown. To help determine the criteria for patient isolation, we evaluated the duration of shedding of Omicron variant virus isolated from upper respiratory samples collected from the reported case-patients in Japan.

This study was approved by the ethics committee of the National Center for Global Health and Medicine (approval no. NCGM-G-003472–03) and the Medical Research Ethics Committee of the National Institute of Infectious Diseases (NIID) for the use of human subjects (approval no. 1178). We obtained written informed consent to publish the article.

## The Study 

We conducted our retrospective study on leftover clinical samples collected from Omicron-infected patients in Japan during November 29–December 18, 2021. We sequenced the Omicron variant by using whole-genome sequencing as described (*2*) and uploaded the consensus sequences to GISAID (https://www.gisaid.org) (Table).

**Table:** **.** Overview of 18 cases of SARS-CoV-2 infection caused by the Omicron variant, Japan, November 29–December 18, 2021*

Case no.	Patient age, y/sex	Disease severity	Vaccine, no. doses (type)	Duration of symptoms, d	Lowest Cq values (days after diagnosis, days after symptom onset)	Virus isolation, since diagnosis (days)‡
1	39/M	Mild	2 (M, M)	5	21.6 (3, 3)	Positive (3)
2	30/M	Asymptomatic	2 (M, M)	NA	25.3 (5, NA)	Positive (5)
3	25/M	Mild	2 (P, P)	6	23.2 (4, 3)	Negative
4	46/M	Mild	3 (J, P, P)	11	24.7 (9, 11)	Positive (6)
5	50/M	Asymptomatic	2 (P, P)	NA	23.1 (5, NA)	Positive (5)
6	31/M	Mild	2 (P, P)	5	25.4 (0, 0)	Negative
7	47/M	Asymptomatic	2 (P, P)	NA	34.2 (9, NA)	Negative
8	33/F	Mild	2 (M, M)	12	32.4 (0, 1)	Negative
9	64/M	Mild	2 (P, P)	4	23.9 (0, −1)	Positive (0)
10	42/M	Mild	2 (M, M)	4	27.0 (0, −1)	Negative
11	49/M	Mild	2 (M, M)	5	26.5 (0, −1)	Positive (8)
12	31/M	Mild	2 (M, M)	4	25.4 (5, 4)	Positive (7)
13	50/M	Mild	2 (M, M)	6	24.7 (5, 7)	Positive (5)
14	30/F	Mild	2 (M, M)	11	30.0 (0, 2)	Negative
15	27/M	Mild	2 (P, P)	8	25.8 (6, 10)	Negative
16	23/M	Mild	2 (P, P)	5	18.7 (3, 4)	Positive (3)
17	47/M	Mild	2 (M, M)	6	24.2 (7, 7)	Positive (0)
18	38/M	Mild	2 (P, P)	6	29.0 (7, 8)	Negative

*The consensus sequences of the viral genome have been uploaded to GISAID (https://www.gisaid.org) (identification nos. EPI_ISL_6913953, EPI_ISL_6914908, EPI_ISL_7194610,　EPI_ISL_7834392,　EPI_ISL_7860184, EPI_ISL_7860185, EPI_ISL_7860188, EPI_ISL_7860189, EPI_ISL_7860190, EPI_ISL_7860193, EPI_ISL_7860197, EPI_ISL_7889642, EPI_ISL_7889643, EPI_ISL_8096984, EPI_ISL_8096995, EPI_ISL_8605240, EPI_ISL_8605241, EPI_ISL_8605242). Cq, quantification cycle; J, Johnson & Johnson; M, Moderna; NA, not available; P, Pfizer/BioNTech; SARS-CoV-2, severe acute respiratory syndrome. coronavirus 2.

For cases detected by SARS-CoV-2 testing at airport quarantines, samples collected for diagnosis (saliva or nasopharyngeal) were transported to the NIID to confirm Omicron. We used the residual samples for this study. The date of sample collection of the first Omicron-positive sample for each patient was defined as the diagnosis date (day 0). Nasopharyngeal samples were collected serially during hospitalization, stored at −80°C, and transported to NIID.

We quantified SARS-CoV-2 RNA by using quantitative reverse transcription PCR (qRT-PCR) and virus isolation testing. We performed qRT-PCR as described previously (*6*). We measured Cq values (i.e., viral RNA levels) by using qRT-PCR targeting the SARS-CoV-2 nucleocapsid gene (Appendix Figure 1). We analyzed samples with Cq values that were reported as negative after 40 cycles by substituting a value of 45. We performed the virus isolation assay according to described procedure (*7*). All laboratory analyses were performed at the NIID.

To examine infectious virus shedding, we classified samples according to date of diagnosis, date of symptom onset, and date of symptom resolution. For cases in which multiple samples were collected in each time segment, we used the sample with the highest amount of viral RNA (i.e., lowest Cq values) in each time segment for each case for comparison. For data analysis and visualization, we used GraphPad Prism version 8.4.3 (https://www.graphpad.com). To compare the Cq values, we used Mann-Whitney *t* and Friedman tests with Dunn multiple comparisons. Statistical significance was set at p<0.05.

All 18 case-patients had been vaccinated >14 days before coronavirus disease (COVID-19) diagnosis (Table). The median (interquartile range [IQR]) duration between vaccination and diagnosis was 117 (71–131) days. Of the 18 case-patients, 15 were symptomatic and 3 were asymptomatic.

**Figure 1:**
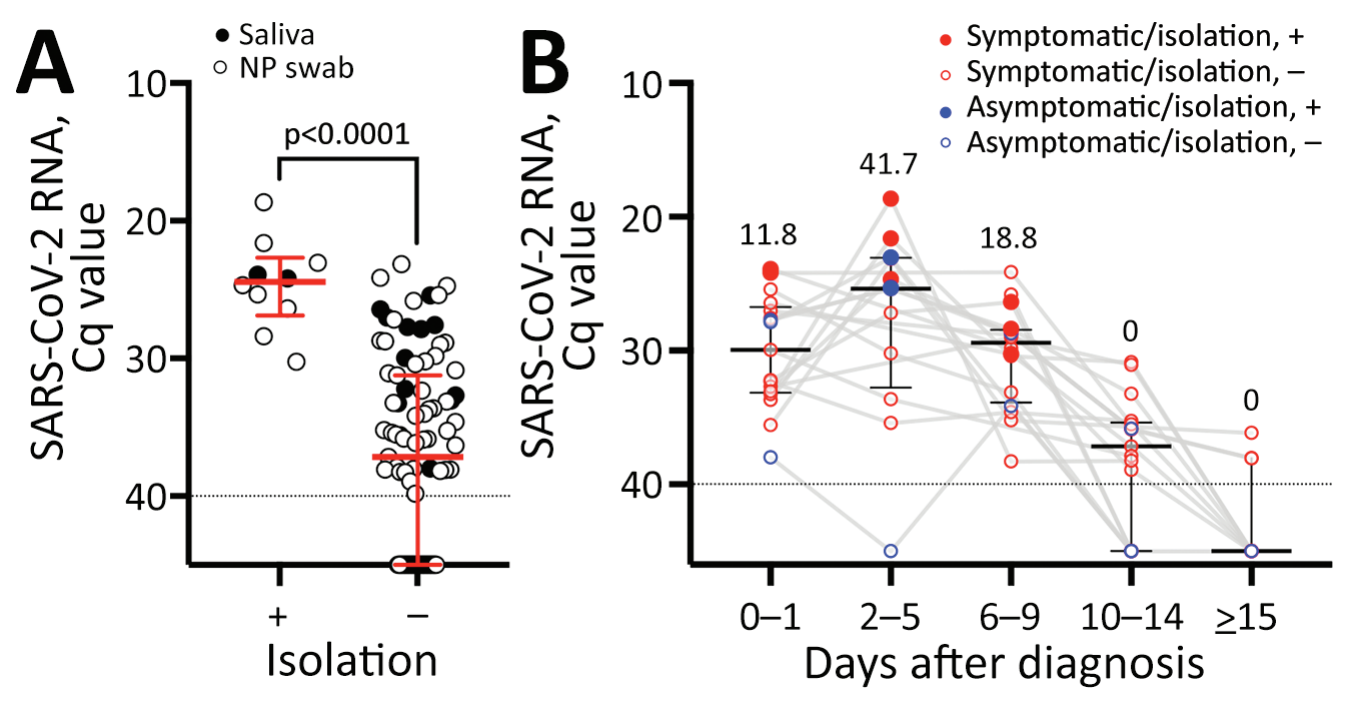
SARS-CoV-2 RNA level and infectious virus shedding in all upper respiratory samples from patients infected with the SARS-CoV-2 Omicron variant, Japan, November 29–December 18, 2021. A) SARS-CoV-2 RNA levels in NP swab samples (open circles) and saliva (closed circles) with or without infectious virus. Red lines indicate median Cq values and error bars interquartile ranges; dotted lines indicate negative cutoff values. The Cq values between samples from which infectious virus was isolated and samples from which infectious virus was not isolated were compared by using the Mann-Whitney test. B) SARS-CoV-2 RNA levels and presence of infectious virus organized by the days after diagnosis. Red circles indicate symptomatic case-patients; blue circles indicate asymptomatic case-patients; each closed circle indicates case-patients from whom virus was isolated. Numbers above each plot indicate the proportion of case-patients from whom virus was isolated in each period. Black lines indicate median Cq values and error bars interquartile ranges; dotted lines indicate negative cutoff values. Cq, quantification cycle; NP, nasopharyngeal; SARS-CoV-2, severe acute respiratory syndrome coronavirus 2; +, with infectious virus; –, without infectious virus.

**Figure 2:**
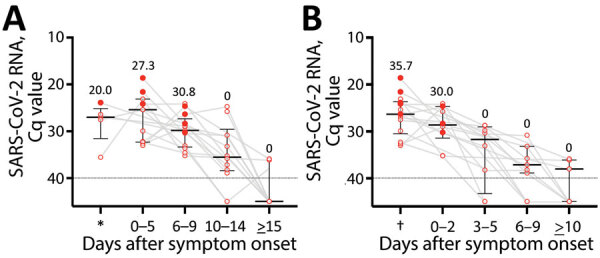
SARS-CoV-2 RNA level and infectious virus shedding in upper respiratory samples from symptomatic patients infected with the SARS-CoV-2 Omicron variant, Japan, November 29–December 18, 2021. A) SARS-CoV-2 RNA levels and presence of the infectious virus, by date of symptom onset. Each closed circle indicates case-patients from whom virus was isolated. Numbers above each plot indicate the proportion of case-patients from whom virus was isolated in each period. Black lines indicate median Cq values and error bars interquartile ranges; dotted lines indicate negative cutoff values. *Before symptom onset. B) SARS-CoV-2 RNA levels and presence of infectious virus, by date of symptom resolution. Closed circles indicate patients from whom virus was isolated. Numbers above each plot indicate the proportion of persons from whom virus was isolated in each period. Black lines indicate median Cq values and error bars interquartile ranges; dotted lines indicate cutoff values. †Before symptom resolution. Cq, quantification cycle; SARS-CoV-2, severe acute respiratory syndrome coronavirus 2.

Among the 101 serially collected samples analyzed (85 nasopharyngeal and 16 saliva), we detected infectious virus in 10 (9.9%) from 10 patients (8 symptomatic and 2 asymptomatic) (Figure 2, panel A; Appendix Tables 1, 2,). The viral RNA levels analyzed by using qRT-PCR were significantly higher in samples with the infectious virus than without (p<0.0001) (Figure 1, panel A). Infectious virus was detected up to 9 days after diagnosis; the highest proportion of virus isolates (41.7%) was found in samples collected 2–5 days after diagnosis, and no isolates were detected 10 days after diagnosis (Figure 1, panel B; Appendix Figure 3, panel A).

We detected infectious virus in the samples of 20%–30% symptomatic patients, ranging from before they were symptomatic to 9 days after symptom onset, but we detected no infectious virus beyond 10 days after symptom onset (Figure 2, panel A; Appendix Table 3, Figure 2, panel B, Figure 3, panel B). For ≈30% of case-patients, infectious virus shedding was detected up to 2 days after symptom resolution, but no virus was detected beyond 3 days after symptom resolution (Figure 2, panel B; Appendix Table 4, Figure 3, panel C). Many of the first samples collected were saliva samples. Of note, the results of only nasopharyngeal samples did not differ from samples including saliva after 2 days of diagnosis (Appendix Figure 4, panels A, B).

## Conclusions 

Omicron RNA detection was highest 2–5 days after diagnosis or after symptom onset and then decreased over time, markedly 10 days after diagnosis or symptom onset. In symptomatic case-patients with infectious virus detected on days 6–9 after symptom onset, infectious virus was also detected 0–2 days after symptom resolution. Although the sample size used in our study is small, these findings suggest the possibility of changes in the viral replication kinetics, unlike previous reports for ancestral (wild-type) strain (Wu01) strains (*8*,*9*). Cq values were frequently lower for the B.1.617.2 (Delta) variant than for the other variants (B.1.1.7 [Alpha]), and virus clearance was faster in vaccinated than in unvaccinated persons (*10*). Similar to findings for the Wu01 strain, the Alpha variant, and the Delta variant (*11*–*13*), RNA of the Omicron variant was detectable 10 days after diagnosis or symptom onset, but no virus was isolated.

In the United States, the isolation period for COVID-19 patients is 5 days after symptom onset if the symptoms are improving (*14*). In Japan, based on the outbreak situation, the results of this study, and isolation criteria in other countries, the isolation criteria for Omicron patients were changed on January 6, 2022. Two consecutive negative test results 10 days after diagnosis or symptom onset are no longer required for patients who received 2 vaccine doses.

Our first study limitation is that we identified infectious virus by infection assays among only 18 patients. We do not know about the infectivity outside of this study. In addition, there are no epidemiologic data about whether secondary infections occurred from patients with these infectious viruses. Therefore, comparing theses results with future epidemiologic studies of more samples is necessary. Our second study limitation is that the virus isolation and infectivity assay results depend on the sample collection method, storage period, and storage conditions. Therefore, negative results do not guarantee that there was no infectious virus in the sample at the time of collection. Last, for some case-patients, virus was not isolated in samples collected at the time of diagnosis. For these persons, the samples used for diagnosis were collected at the airport quarantine and were saliva, for which the quality may not be suitable for virus isolation. Although our results are insufficient to show a difference in efficiency of virus isolation between saliva and nasopharyngeal samples in Omicron-infected persons, this difference may have underestimated the presence of infectious virus at diagnosis. In conclusion, fully vaccinated COVID-19 case-patients with mild or asymptomatic infection shed infectious virus in their upper respiratory tract for 6–9 days after illness onset or diagnosis, even after symptom resolution, but not after day 10.

Appendix Supplemental results from study of duration of infectious virus shedding by SARS-CoV-2 Omicron variant–infected vaccinees, Japan, November 29–December 18, 2021.
