# Supplementary material for: Duration of Infectious Virus Shedding by SARS-CoV-2 Omicron Variant–Infected Vaccinees
Source: Emerg Infect Dis. 2022 May;28(5):998–1001. doi: 10.3201/eid2805.220197 (PMC9045443; doi:10.3201/eid2805.220197)
Supplement: Appendix — Supplemental results from study of duration of infectious virus shedding by SARS-CoV-2 Omicron variant–infected vaccinees, Japan, November 29–December 18, 2021. [file 22-0197-Techapp-s1.pdf]

Article DOI: https://doi.org/10.3201/eid2805.220197

Duration of Infectious Virus Shedding by SARS-CoV-2 Omicron Variant–Infected Vaccinees

Appendix

Appendix Table 1. SARS-CoV-2 RNA-positivity / viral isolation-positivity of SARS-CoV-2 Omicron variant-infected cases, organized by days post diagnosis (all cases)

Days post diagnosis	Positive for SARS-CoV-2 RNA	Cq value	SARS-CoV-2 virus isolation
0–1 days	17/17 (100)	30.0 (27.0-33.1)	2/17 (11.8)
2–5 days	11/12 (91.7)	25.4 (23.1-31.1)	5/12 (41.7)
6–9 days	16/16 (100)	29.4 (28.6-33.4)	3/16 (18.8)
10–14 days	12/17 (70.6)	37.2 (35.5-45.0)	0/17 (0)
After 15 days	3/10 (30.0)	45.0 (39.8-45.0)	0/10 (0)

*Unless otherwise stated, data are presented as n (%). Cq values are presented as the median (interquartile range). SARS-CoV-2, severe acute respiratory syndrome coronavirus 2; RNA, ribonucleic acid.

Appendix Table 2. SARS-CoV-2 RNA-positivity / viral isolation-positivity of SARS-CoV-2 Omicron variant-infected cases, organized by days post diagnosis (asymptomatic cases)*

Days post diagnosis	Positive for SARS-CoV-2 RNA	Cq value	SARS-CoV-2 virus isolation
0–1 days	3/3 (100)	27.7, 27.9, 38.0	0/3 (0)
2–5 days	2/3 (66.7)	23.1, 25.3	2/3 (66.7)
6–9 days	2/2 (100)	28.7, 34.2	0/2 (0)
10–14 days	1/3 (33.3)	35.8	0/3 (0)
After 15 days	0/1 (0)	NA	0/1 (0)

*Unless otherwise stated, data are presented as n (%). Cq values are presented as an actual number. SARS-CoV-2, severe acute respiratory syndrome coronavirus 2; RNA, ribonucleic acid; NA, not available.

Appendix Table 3. SARS-CoV-2 RNA-positivity / viral isolation-positivity of SARS-CoV-2 Omicron variant-infected cases, organized by days post symptom onset (symptomatic cases only)*

Days post diagnosis	Positive for SARS-CoV-2 RNA	Cq value	SARS-CoV-2 virus isolation
Presymptomatic	5/5 (100)	27.0 (26.5-27.6)	1/5 (20.0)
0–5 days	11/11 (100)	25.4 (23.7-32.3)	3/11 (27.3)
6–9 days	13/13 (100)	29.8 (28.4-33.1)	4/13 (30.8)
10–14 days	11/13 (84.6)	35.5 (31.1-38.2)	0/13 (0)
After 15 days	3/10 (30.0)	45.0 (39.7-45.0)	0/10 (0)

*Unless otherwise stated, data are presented as n (%). Cq values are presented as the median (interquartile range). SARS-CoV-2, severe acute respiratory syndrome coronavirus 2; RNA, ribonucleic acid.

Appendix Table 4. SARS-CoV-2 RNA-positivity / viral isolation-positivity of SARS-CoV-2 Omicron variant-infected cases, organized by days post symptom resolution (symptomatic cases only)*

Days post symptom resolution	Positive for SARS-CoV-2 RNA	Cq value	SARS-CoV-2 virus isolation
Before	14/14 (100)	26.4 (24.0-29.4)	5/14 (35.7)
0–2 days	10/10 (100)	28.7 (25.0-30.2)	3/10 (30.0)
3–5 days	10/12 (83.3)	32.2 (30.3-38.0)	0/12 (0)
6–9 days	6/9 (66.7)	38.2 (35.8-45.0)	0/9 (0)
After 10 days	3/8 (37.5)	45.0 (37.6-45.0)	0/8 (0)

*Unless otherwise stated, data are presented as n (%). Cq values are presented as an actual number. SARS-CoV-2, severe acute respiratory syndrome coronavirus 2; RNA, ribonucleic acid; NA, not available.

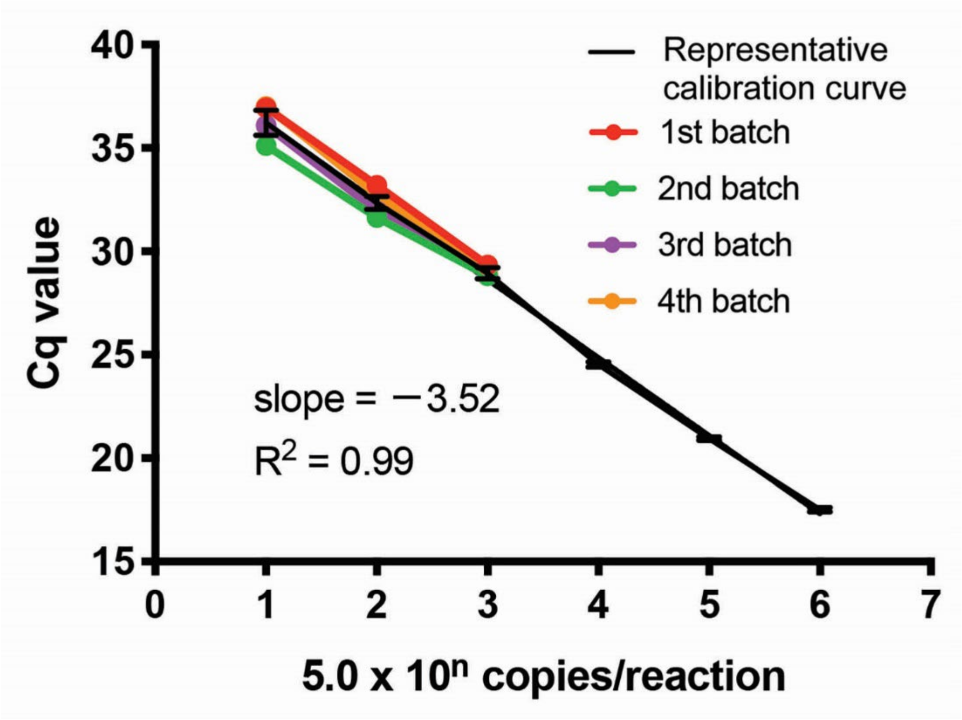

Appendix Figure 1. Perfomance characteristics of the SARS-CoV-2 RT-qPCR used in this study. All of the SARS-CoV-2 RT-qPCR assays in this study were performed at the NIID using One Step PrimeScript III RT-qPCR Mix (Takara Bio, Shiga, Japan), the N2 primer/probe set (NIID_2019-nCoV_N_F2 primer, NIID_2019-nCoV_N_R2 primer, and NIID_2019_nCoV_N_P2 probe), and a Roche LightCycler 96 (Roche, Basel, Switzerland). The Cq value of each sample in this study was acquired from four independently performed assays. For each assay, 50 copies, 500 copies, 5,000 copies of quantified in vitro transcribed RNA were included as control samples to ensure the integrity of the assay. The graph shows an overlay of a representative calibration curve for the assay with the quantified in vitro transcribed RNA samples and the Cq values of the control samples from each assay. The Cq values of the control samples in each assay fitted the representative calibration curve well, and the deviation of the Cq values of the control samples in each assay was minimal.

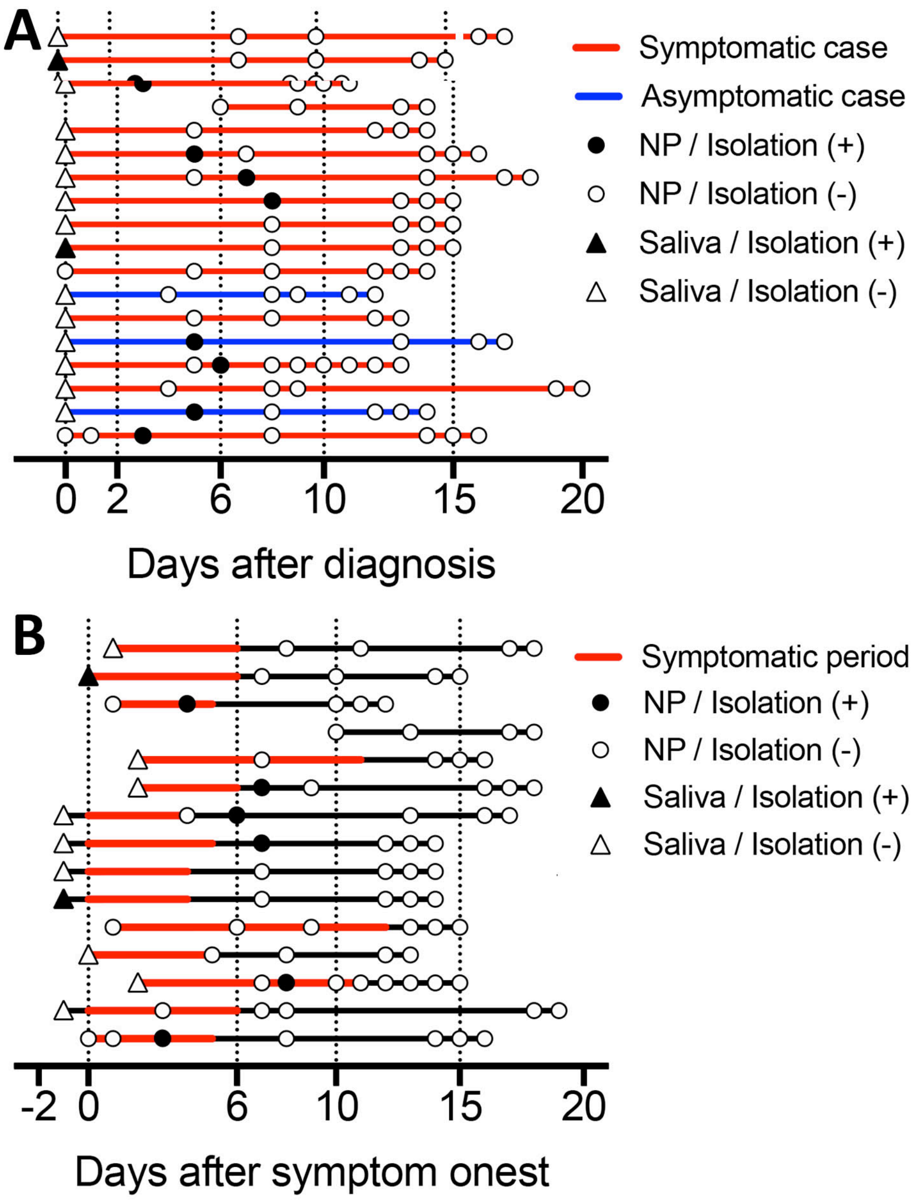

Appendix Figure 2. The timeline of upper respiratory samples collected from individuals infected with the SARS-CoV-2 Omicron variant (A and B) The timeline of collection of nasopharyngeal (NP) swabs (circles) and saliva (triangles) from individuals infected with the SARS-CoV-2 Omicron variant is indicated as horizontal line for each case. (A) The timeline starts from the day of diagnosis and extends to each sample collected from each case. Each symbol (circle for NP, or triangle for saliva) indicates that a sample was collected. Closed symbol: infectious virus (+); open symbol: infectious virus (-); red line:

symptomatic case; blue line: asymptomatic case. (B) The timeline starts from the day of symptom onset and extends to each sample collected from each case. Each symbol Each symbol (circle for NP, or triangle for saliva) indicates that a sample was collected. Closed symbol: infectious virus (+); open symbol: infectious virus (-). Red line: symptomatic period.

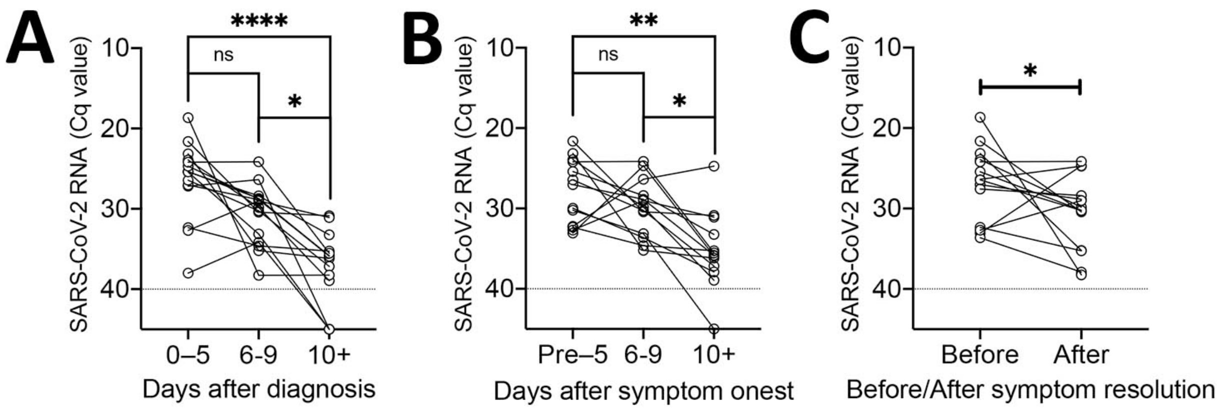

Appendix Figure 3. Comparison between Cq values and days after dianosis, days after symptom onset, before/after symtom resolution (A) The levels of SARS-CoV-2 RNA at the time periods after diagnosis were compared. The Cq values among samples in each timepoint were compared using Friedman test with Dunn's multiple comparisons test; ****p < 0.0001, *p < 0.05, ns; not significant. (B) The levels of SARS-CoV-2 RNA at the time periods before or after symtom onset were compared. The Cq values among samples in each timepoint were compared using Friedman test with Dunn's multiple comparisons test; **p < 0.01, *p < 0.05, ns; not significant. (C) The levels of SARS-CoV-2 RNA at the time periods before or after symtom resolution were compared. The Cq values among samples in each timepoint were compared using Mann Whitney test; *p < 0.05.

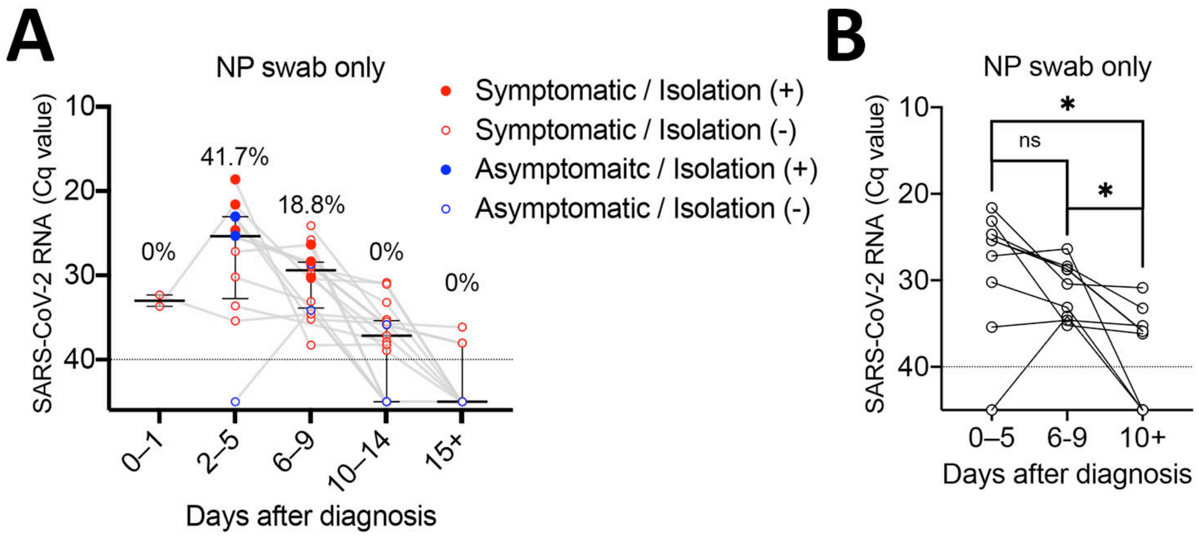
A
NP swab only
Symptomatic / Isolation (+)
Symptomatic / Isolation (-)
Asymptomaitc / Isolation (+)
Asymptomatic / Isolation (-)
SARS-CoV-2 RNA (Cq value)
10
20
30
40
0%
41.7%
18.8%
0%
0%
0–1
2–5
6–9
10–14
15+
Days after diagnosis
B
NP swab only
SARS-CoV-2 RNA (Cq value)
ns
*
*
0–5
6-9
10+
Days after diagnosis
